# The Influence of Self-Esteem and Psychological Flexibility on Medical College Students' Mental Health: A Cross-Sectional Study

**DOI:** 10.3389/fpsyt.2022.836956

**Published:** 2022-05-16

**Authors:** Jiamei Guo, Xin Huang, Anhai Zheng, Wanjun Chen, Zhongli Lei, Chenglu Tang, Hongyu Chen, Hongyan Ma, Xuemei Li

**Affiliations:** ^1^Department of Psychiatry, The First Affiliated Hospital of Chongqing Medical University, Chongqing, China; ^2^Department of Psychology, Hunan University of Medicine, Hunan, China

**Keywords:** self-esteem, psychological flexibility, mental health, empirical avoidance, cognitive fusion

## Abstract

**Background:**

Mental health problems has become a major public health issue among medical students. Self-esteem and psychological flexibility were important associated factors for mental health, but their relations have not been discussed in medical students. The present study aimed to assess the status of mental health problems among medical students and identified whether psychological flexibility had a mediating role in the effects of self-esteem on the top three most common psychological symptoms.

**Methods:**

A total of 810 undergraduate students from 18 classes comprised in the sample. Nine dimensions of psychological symptoms was assessed by the Symptom Checklist-90-revised (SCL-90-R). Self-esteem was measured by the Self-esteem Scale (SES) and psychological flexibility was evaluated by the Acceptance and Action Questionnaire 2nd Edition (AAQ-II) and Cognitive Fusion Questionnaire (CFQ-F). Univariate analysis and logistic regression analysis were used to determine the relationship among the top three common psychological symptoms, self-esteem, psychological flexibility, and participants' characteristics. The mediating effect of psychological flexibility between self-esteem and psychological symptoms was detected by bootstrap method.

**Results:**

57.8% of the medical undergraduate students reported positive at least one of the nine psychological symptom dimensions assessed by the SCL-90-R and 13.8% of students had moderate or more severe symptoms. The symptoms of obsessive-compulsiveness, interpersonal sensitivity, and depression were the three most common psychological symptoms among the medical students. Meanwhile, self-esteem and psychological flexibility were negatively associated to the symptoms of obsessive-compulsiveness, interpersonal sensitivity, and depression. And, almost 50% effects of self-esteem on these three symptoms in medical students exert indirect effects through psychological flexibility.

**Conclusions:**

Psychological distress was quite common in the Chinese medical students. The three most common psychological symptoms were successively obsessive-compulsiveness, interpersonal sensitivity, and depression. Low self-esteem and psychological inflexibility might be the risk factors for these top three symptoms, and psychological flexibility might play a mediating role in the effects of self-esteem on these psychological symptoms.

## Introduction

Mental health is an essential component of health (1)_._ Yet, mental health problems have become a major public health issue worldwide due to its increasing prevalence and great burden to their own families or the society (2, 3). Most mental health problems arise in early adulthood. However, young adults rarely receive any treatment or support from professionals (4). It is reported that college students are facing greater mental health challenges than general population due to high expectations of families and society (5, 6). Worldwide, it is estimated that 12%−50% of college students present at least one diagnostic criterion for one or more mental disorders (7). And even more, medical students are at a higher rate of mental health problems than non-medical undergraduates, such as anxiety, depression, stress, suicide and so on (8). These problems might lead to a range of adverse effects, such as poor academic performance, bad relationships with peers, and increasing risk of suicide and non-suicidal self-injury (NSSI) behavior (9, 10). Moreover, they might also potentially increase the incidence of drug abuse, medical errors, and unethical behavior after graduation (11). Therefore, early identification and management of psychological distress may be critical for long-term knowledge accumulation and development of medical students as future health care professionals.

Studies have suggested that self-esteem plays an important role in mental health (12). Self-esteem is an overall self-evaluation of an individual's worth and ability, strongly affected by social feedback from parents, teachers, and peers (13). It influences interactions and feelings toward oneself and others. Strong evidence shows that low self-esteem is a non-specific risk factor for mental health, such as anxiety, depression, stress or aggression (14). Choi et al. (15) suggested that different levels of self-esteem was related to the development of depression, and also related the social support. Meanwhile, Self-esteem is also associated to substance abuse, suicidal ideation, suicidal attempted, and other mental health conditions (16, 17). Therefore, improving the level of self-esteem is helpful to promote the mental health for young adults.

Psychological flexibility, a fundamental aspect of health (18), is the central construction of Acceptance and Commitment Therapy (ACT) proposed by Hayes et al. (19). Psychological flexibility is defined as an individual's “ability to contact with the present environment and internal psychological activities, to persist or change in behavior when doing so serves valued ends, and to tolerate, accept, or overcome interference (19). Empirical avoidance and cognitive fusion are the features of psychological inflexibility. Experiential avoidance refers to an unwillingness to maintain contact with unwanted experiences and acting to avoid them (20), and cognitive fusion refers to the tendency for behavior to be over-regulated and affected by cognition (21). Psychological flexibility has been found to be closely associated with psychopathology and wellbeing (22). Apart for that, psychological flexibility also mediate the development of mental disorders. Over the past two decades, ACT have gained a amount of attention in clinical psychotherapy and was widely used in the treatment of anxiety, depression, chronic physical pain, and so on (23–25). The core treatment target in ACT is to promote psychological flexibility. A high level of psychological flexibility can enhance a positive evaluation of oneself, feelings of continuous growth and development, purposeful and meaningful beliefs in life, and satisfaction with one's relationship (26). To some extent, these are all closely related to self-esteem. Hence, we speculated that the level of self-esteem may be improved by an increased psychological flexibility.

As mentioned above, both self-esteem and psychological flexibility are important related factors for mental health. However, so far, no studies have been reported on the relations between self-esteem, psychological flexibility, and mental health problems. Self-esteem, as a central part of human personality, whether it can modulate mental health by mediating psychological flexibility is unknown. So, in the study, we would explore the link between self-esteem, psychological flexibility, and mental health problems, and further confirm whether psychological flexibility had a mediating effect between self-esteem and mental health. Although, there have been some studies on the prevalence of and risk factors for mental health problems among medical students (27, 28), but little is known about the role of psychological flexibility on the mental health problems in this specific group. Secondly, most studies focused on the incidence of depression, anxiety, stress and their related factors, and little attention was paid to other psychological symptoms. Therefore, our study would also clarify the status of psychological symptoms among medical students, and analyze the related risk factors for the top three psychological symptoms respectively. Our findings might be helpful to provide valuable information for mental health services in medical student.

## Methods

### Subjects and Study Design

A Cross-sectional study was conducted in Chongqing, a major city in southwest China during June 1 to December 31, 2019. Participants were medical students from Chongqing medical university. A stratified cluster sampling method was used to draw a 25% sample of undergraduate students in the university. All the students in the chosen class were enrolled into the study. With this sampling procedure, a total of 810 medical university students from 18 classes comprised the study population. All the subjects were informed about the purpose of the study, the confidentiality of personal information, the principle of volunteering and written informed consent was obtained from all study participants before evaluation. Study protocols were approved by the ethics committee of Chongqing Medical University, China.

### Measurement Process

Data collection was conducted by two trained members of the research group. Before the assessments, the testers introduced the purpose and significance of the survey and declared the principle of confidentiality to all the participants. After obtaining the students' informed consent, questionnaires were distributed centrally and unified instructions were informed. The participants answered the questionnaires independently and anonymously. During the assessments, the testers were always on the spot to answer any questions raised by the participants. The answer time for the questionnaires was within 30 min. Psychological assessments were composed of four parts: demographics, self-esteem assessments, psychological distress, and psychological flexibility. Before assessing each scale, identified instructions were provided.

### Measures

#### Demographics

General information covered age, gender, grades, registered residence (urban or rural), relationship with roommates (good, not bad, bad), being the only child or not, being a poor student or not, joining the student clubs or not.

#### Symptom Checklist-90-Revised Scale (SCL-90-R)

Psychological symptoms of study participants were assessed using the Chinese version of the Symptom Checklist-90-revised (SCL-90-R), which was widely used in mental health screening (29). The scale has a total of 90 items and is divided into nine dimensions for evaluating participants' self-reported psychopathological symptoms, including somatization, obsessive-compulsiveness, interpersonal sensitivity, depression, anxiety, hostility, phobic anxiety, paranoid ideation, and psychoticism. Each item is scored using a 5-point Likert-type scale according to the severity to which the subjects had suffered from the item in the last week (0 = “not at all”, 1 = “a little bit”, 2 = “moderately”, 3= “quite a bit”, 4 = “extremely”). Higher scores indicate a greater severity of psychiatric symptoms. The scores of the nine subscales were categorized as follows: average score <1 were classified as “normal”; 1 ≤ average score < 2 were classified as “mild”; and average score ≥2 were classified as “moderate or high”. In the present study, the Cronbach's alpha coefficient for this scale was 0.975 and ranged from 0.746 (paranoid ideation) to 0.904 (depression) across the nine subscales.

#### Self-Esteem Scale

Self-esteem Scale (SES) was compiled by Rosenberg in 1965. The Chinese version of self-esteem scale was used to measure the self-esteem levels (30). It consists of 10- item self-reported questionnaire with a 4-point scale. The questions are about participants' overall feelings of self-worth and self-acceptance. The total scores range from 10 to 40, with the higher scores indicating higher self-esteem. In this survey, the Cronbach coefficient of the scale in the present research was 0.836.

#### Psychological Flexibility Assessments

Psychological flexibility was assessed *via* the Chinese version of Acceptance and Action Questionnaire 2nd Edition (AAQ-II) and Cognitive Fusion Questionnaire (CFQ-F). AAQ-II was used to assess experiential avoidance using a 7-item self-reported questionnaire with a 7-point scale (31). The total scores range from 7 to 49, with the higher scores indicating greater psychological inflexibility. CFQ-F was used to measure cognitive fusion using a 9-items self-reported questionnaire with a 7-point scale (32). The total scores range from 9 to 63, with the higher scores indicating higher psychological inflexibility. In this study, the Cronbach coefficient of the AAQ-II and CFQ-F questionnaires were 0.883 and 0.946, respectively.

### Statistical Analysis

Data were analyzed in IBM SPSS Statistics 22.0. Descriptive statistics was applied to the study variables and general characteristics. Univariate analyses were used to identify any relations between sociodemographic characteristics, self-esteem, psychological flexibility and the psychological symptoms. Additionally, linear regressions analysis was constructed to analyze the relationship between psychological symptoms and self-esteem and psychological flexibility. Bootstrap procedure was used to explore the mediating effects of psychological flexibility on the self-esteem and psychological symptoms. *P* (two-tailed) < 0.05 was considered statistically significant.

## Results

In total, 810 questionnaires were distributed to medical students, and 788 valid questionnaires were used for the next analysis. Hence, the effective rate of valid data received for the present analysis was 97.28%. The participants were aged between 17 and 24 years, with an average age of 19.93 (SD = 1.27), and 60.7% of the participants were female. Among them, the proportions of freshmen, sophomores, and juniors 33.9, 34.1, and 32.0%, respectively. Most participants were rural residents (*n* = 537; 68.1%), and had one or more siblings (*n* = 607; 77%). 81.9% of the participants had a good relationship with their roommates, and only 13.2 of participants were poor students (Table 1).

**Table 1:** The study sample description (*N* = 788).

**Sociodemographic**	**Total (*N* = 788)**
**Age (years), Mean (SD)**	19.931 ± 0.27
**Residence**, ***n*** **(%)**
Urban	250 (31.7)
Rural	537 (68.1)
**Sex**, ***n*** **(%)**
Male	310 (39.3)
Female	478 (60.7)
**Grades**, ***n*** **(%)**
First	267 (33.9)
Second	269 (34.1)
Third	252 (32)
**Being the Only child or not**, ***n*** **(%)**
Yes	181 (23)
No	607 (77)
**Joining a student club or not**, ***n*** **(%)**
Yes	546 (81.9)
No	136 (17.3)
**Relationships with roommates**, ***n*** **(%)**
Good	645 (81.9)
Not bad	136 (17.3)
Bad	7 (0.9)
**Bing a poor student or not**, ***n*** **(%)**
Yes	104 (13.2)
No	684 (86.8)

Of our participants, 50.5% had symptoms of obsessive-compulsiveness (8.5% with moderate or more severe), 38.1% had symptoms of interpersonal sensitivity (6.9% with moderate or more severe), 26.6% had symptoms of depression (5.7% with moderate or more severe), and 21.6% had symptoms of anxiety (3.3% with moderate or more severe). These psychological symptoms were more common in the medical students. The prevalence of other psychological symptoms is shown in Table 2, such as symptoms of hostility, somatization, phobia, paranoid, and psychoticism. Besides, 57.8% of participants reported positive at least one dimension of symptoms (13.8% with moderate or more severe).

**Table 2:** Distribution of SCL-90-R.

**SCL-90-R**	**Distribution of all participant, *n* (%)**
**Somatization**
Normative	721 (91.5)
Mild	64 (8.1)
Moderate or high	3 (0.4)
**Obsessive-compulsiveness**
Normative	390 (49.5)
Mild	331 (42)
Moderate or high	67 (8.5)
**Interpersonal sensitivity**
Normative	488 (61.9)
Mild	246 (31.2)
Moderate or high	54 (6.9)
**Depression**
Normative	578 (73.4)
Mild	165 (20.9)
Moderate or high	45 (5.7)
**Anxiety**
Normative	618 (78.4)
Mild	144 (18.3)
Moderate or high	26 (3.3)
**Hostility**
Normative	647 (82.1)
Mild	121 (15.4)
Moderate or high	20 (2.5)
**Phobia**
Normative	644 (81.7)
Mild	124 (15.7)
Moderate or high	20 (2.5)
**Paranoid**
Normative	620 (78.7)
Mild	148 (18.8)
Moderate or high	20 (2.5)
**Psychoticism**
Normative	610 (77.4)
Mild	155 (19.7)
Moderate or high	23 (2.9)
**Overall of each dimension**
Normative	332 (42.1)
Mild	347 (44)
Moderate or high	109 (13.8)

Table 3 shows that experiential avoidance and cognitive fusion were positively correlated with the symptoms of obsessive-compulsiveness, interpersonal sensitivity, and depression (*P* < 0.001), and self-esteem was negatively correlated with these three symptoms (*P* < 0.001). Table 3 also shows that age, sex, grades and relationships with roommates were related with symptoms of obsessive-compulsiveness and depression (*P* < 0.05). Additionally, joining a student club was associated with symptoms of depression (*P* = 0.044). But, we only observed grades (*P* = 0.028) and relationships with roommates (*P* < 0.001) was related with the symptoms of interpersonal sensitivity.

**Table 3:** Factors influencing obsessive-compulsiveness, interpersonal sensitivity, and depression symptoms (mean ± SD).

**Sociodemographic**	**Obsessive-compulsiveness**	***r/t/F***	***P***	**Interpersonal sensitivity**	***r/t/F***	***P***	**Depression**	***r/t/F***	***P***
**Age (years)**		0.114	**0.001** ^**c**^		0.061	0.089^c^		0.11	**0.002** ^**c**^
**Place of birth**		−1.003	0.316^a^		−0.635	0.526^a^		0.546	0.585^a^
Urban	1.020 ± 0.65			0.810 ± 0.65			0.700 ± 0.69		
Rural	1.070 ± 0.65			0.840 ± 0.63			0.670 ± 0.62		
**Sex**		−2.197	**0.028** ^**a**^		−1.338	0.181^a^		−2.903	**0.004** ^**a**^
Male	0.990 ± 0.64			0.790 ± 0.64			0.600 ± 0.60		
Female	1.090 ± 0.65			0.860 ± 0.64			0.730 ± 0.67		
**Grades**		10.107	**<0.001** ^**b**^		3.597	**0.028** ^**b**^		9.241	**<0.001** ^**b**^
Freshman year	0.960 ± 0.59			0.780 ± 0.61			0.570 ± 0.57		
Sophomore year	1.010 ± 0.63			0.800 ± 0.64			0.670 ± 0.65		
Junior year	1.200 ± 0.71			0.920 ± 0.66			0.680 ± 0.64		
**Being the only child or not**		−0.937	0.349^a^		−0.223	0.824^a^		−0.301	0.764^a^
Yes	1.010 ± 0.71			0.820 ± 0.71			0.670 ± 0.71		
No	1.060 ± 0.63			0.830 ± 0.62			0.680 ± 0.62		
**Joining a student club or not**		−1.759	0.079^a^		−0.846	0.398^a^		−2.015	0.044^a^
Yes	1.020 ± 0.64			0.810 ± 0.63			0.640 ± 0.59		
No	1.100 ± 0.66			0.850 ± 0.65			0.730 ± 0.70		
**Relationships with roommates**		19.92	**<0.001** ^**b**^		32.42	**<0.001** ^**b**^		42.786	**<0.001** ^**b**^
Good	1.990 ± 0.61			1.750 ± 0.58			1.590 ± 0.55		
Not bad	2.290 ± 0.72			2.140 ± 0.72			2.060 ± 0.82		
Bad	2.961 ± 0.07			2.861 ± 0.24			2.71 ± 0.16		
**Bing a poor student or not**		−1.05	0.294^a^		1.602	0.11^a^		0.984	0.326^a^
Yes	1.110 ± 0.67			0.920 ± 0.71			0.740 ± 0.68		
No	1.040 ± 0.65			0.820 ± 0.63			0.670 ± 0.64		
Empirical avoidance		0.615	**<0.001** ^**c**^		0.643	**<0.001** ^**c**^		0.657	**<0.001** ^**c**^
Cognitive fusion		0.586	**<0.001** ^**c**^		0.587	**<0.001** ^**c**^		0.601	**<0.001** ^**c**^
Self-esteem scale(SES)		−0.466	**<0.001** ^**c**^		−0.527	**<0.001** ^**c**^		−0.588	**<0.001** ^**c**^

a *t-value*.

b *F-value*.

c *r-value*.

*The bold values indicate p < 0.05*.

Linear regression analysis further showed the self-esteem was negatively associated with the symptoms of obsessive-compulsiveness (*B* = −0.024, β = −0.163, *P* < 0.001), interpersonal sensitivity (*B* = −0.034, β = −0.234, *P* < 0.001) and depression (*B* = −0.043, β = −0.292, *P* < 0.001). Table 4 also shows that cognitive fusion and empirical avoidance were positively related with the symptoms of obsessive-compulsiveness (*B* = 0.013, β = 0.25, *P* < 0.001; *B* = 0.025, β = 0.32, *P* < 0.001), interpersonal sensitivity (*B* = 0.01, β = 0.195, *P* < 0.001; *B* = 0.027, β = 0.348, *P* < 0.001) and depression (*B* = 0.01, β = 0.188, *P* < 0.001; *B* = 0.025, β = 0.324, *P* < 0.001) after adjusted for age, sex, grades, roommate relationship, family economic status.

**Table 4:** Linear regression of SES and psychological flexibility for the top three most common symptoms.

**Independent variables**	**Obsessive-compulsiveness score**			**Interpersonal sensitivity score**			**Depression score**		
	***B***	**95% CI**		***B***	**95% CI**		***B***	**95% CI**	
		**Lower**	**Upper**		**Lower**	**Upper**		**Lower**	**Upper**
SES	−0.024	−0.034	−0.015	−0.034	−0.043	−0.026	−0.043	−0.052	−0.035
Empirical avoidance	0.025	0.018	0.032	0.027	0.02	0.033	0.025	0.019	0.031
Cognitive fusion	0.013	0.008	0.017	0.01	0.006	0.014	0.01	0.006	0.013

*After adjusted for age, sex, grades, roommates relationship, family economic status*.

In order to further understand the relationship among self-esteem, psychological flexibility and the top three psychological symptoms in the medical students, we explored whether psychological flexibility has a mediating effect between self-esteem and these three psychological symptoms. First, the predictive variables were standardized. The scores of obsessive-compulsiveness, interpersonal sensitivity, and depression were chosen as the dependent variable, respectively. The scores of SES scores were chosen as the predictive variable. Meanwhile, AQQ-II scores and CFQ-F scores were chosen as the mediating variable to conduct the mediation effect test, and Bootstrap self-sampling times were set as 5,000. The mediating role of psychological flexibility in the effects of self-esteem on the symptoms of compulsive symptoms, obsessive-compulsiveness, and interpersonal sensitivity was −0.286 (experiential avoidance −0.173; cognitive fusion −0.113), −0.278 (experiential avoidance −0.193; cognitive fusion −0.085), and −0.265 (experiential avoidance −0.183; cognitive fusion −0.082), respectively. The proportion of mediation effects ranged from 45.14 to 61.36%, and experience avoidance accounted for a higher proportion of mediation effects compared with cognitive fusion (Table 5).

**Table 5:** Mediation effect of psychological flexibility on self-esteem and the top three most common symptoms.

**Effects**	**Obsessive-compulsiveness**				**Interpersonal sensitivity**				**Depression**			
	**Value**	**Bootstrap 95%** ***CI***		**Relative effect proportion(%)**	**Value**	**Bootstrap 95%** ***CI***		**Relative effect proportion(%)**	**Value**	**Bootstrap 95%** ***CI***		**Relative effect proportion(%)**
		**Lower**	**Upper**			**Lower**	**Upper**			**Lower**	**Upper**	
Total effects	−0.466	−0.528	−0.405	100	−0.527	−0.586	−0.467	100	−0.588	−0.644	−0.531	100
Direct effects	−0.180	−0.243	−0.118	38.64	−0.249	−0.309	−0.189	47.2	−0.322	−0.379	−0.265	54.86
Indirect effects	−0.286	−0.343	−0.236	61.36	−0.278	−0.327	−0.228	52.8	−0.265	−0.316	−0.219	45.14
Empirical avoidance	−0.173	−0.232	−0.118	37.01	−0.193	−0.250	−0.139	36.61	−0.1830	−0.237	−0.134	31.15
Cognitive fusion	−0.114	−0.158	−0.074	24.35	−0.085	−0.127	−0.046	16.19	−0.082	−0.122	−0.049	13.99

## Discussion

To our knowledge, this was the first study to estimate the relationship among self-esteem, psychological flexibility, and mental health in a sample of Chinese medical students. SCL-90 is one of the most famous mental health scales for mental disorders screening in the world (33). This scale is mainly used to assess whether a person has psychological symptoms and its severity, and it has a good discriminatory ability for people with psychological symptoms (34). In our study, we found that psychological distress was a serious problem among Chinese medical students, with 57.8% of medical students reported positive of psychological symptoms, including 44% reporting mild and 13.8% reporting moderate or more severe psychological distress in at least one dimension assessed by SCL-90-R. Our findings indicated that a significant proportion of medical students are experiencing a variety of psychiatric symptoms, which was consistent with previous reported domestic and foreign studies. For example, Yang et al. (27) reported that 24.45% of medical students in southeast China might have mental health problems according to the total score of SCL-90 (≥160). Tang et al. showed that 40.7% of the university students screened positive for at least one of the nine psychological symptom dimensions assessed by the SCL-90-R (35). Shao et al. (3) used Zung self-rating depression scale and Zung Self-Rating Anxiety Scale to evaluate the depression and anxiety symptoms among Chinese medical students, and found that 57.5 and 30.8% of students might be experiencing the symptoms of depression and anxiety, respectively. Meanwhile, foreign researches also find that medical students have a high prevalence of mental health problems, and multiple countries have proposed it is time to act mental health services for medical students (36). Such as a study from Brazilian reported that 34.6% of medical students had depressive symptoms, 37.2% showed anxiety symptoms, and 47.1% had stress (37). Another study from Nigeria showed that 25.2% of the medical students had psychological distress, depression in 33.5%, anxiety in 28.8%, and psychoactive substance in up to 44.2% (38). However, we also noticed that there were some inconsistencies in the reported prevalence rates. These discrepancies might be partly explained by the unbalanced economic development between different regions, different cultures, varied screening methods and different population.

Our present study also found that obsessive-compulsive symptoms were most prevalent among medical students, followed by interpersonal sensitivity and depression. This finding was consistent with previous study reported by Yang (22) using the same scale (SCL-90) to assess the mental health of medical students in Huazhong University of Science and Technology, China. However, Yang's study just simply reported the prevalence of the psychological symptoms (22). They didn't examine the associated factors for the three top psychological symptoms, respectively. To our knowledge, this was the first study to examine the related factors of the three most common psychological symptoms among medical students. Previous studies have also shown that these three psychiatric symptoms seriously affect the mental health of medical students. For example, Esan et al. reported that 51.4% were aware of obsessive-compulsive disorder based on Yale-Brown obsessive compulsive scale(Y-BOCS) among 1,172 medical students (39). Another cross-sectional study with 471 Brazilian medical students found that possible obsessive-compulsive disorder (OCD) identified by Obsessive-Compulsive Inventory-Revised score >27 among medical students is more common (40). Moreover, obsessive-compulsive symptoms were also relevant to suicidal ideation and depressive symptoms among medical students (41). Interpersonal sensitivity is a type of personality trait, characterized by over-awareness of the actions and feelings of others and sensitivity to perceived criticism or rejection (42). Individuals with this trait have a sense of discomfort and inferiority in interpersonal communication, which leads the individuals to often avoid social interactions (43). Interpersonal sensitivity plays an important role in human mental health, and it has some negative impact on individual mental health and social adaptive functions (44). Additionally, it is also a susceptibility factor for many mental disorders such as depression, anxiety, paranoia, and compulsion (45, 46). Thus, obsessive-compulsiveness and interpersonal sensitivity are negligible mental health problems among medical students. Unfortunately, the current domestic and foreign researches on the mental health mainly focused on professional burnout, depression, suicide and anxiety (47). The attention paid to obsessive-compulsiveness and interpersonal sensitivity is still insufficient. Therefore, comprehensive assessment of individual mental health might be more conducive to meet the requirements of WHO for mental health. We considered that screening and intervention for the symptoms of obsessive-compulsive and interpersonal sensitivity should be emphasized in the studies and mental health care among medical students.

In addition, the present study also found a negative association all existed between self-esteem and obsessive-compulsiveness, interpersonal sensitivity, or depression. It means that low self-esteem was a risk factor for these three psychological symptoms. Self-esteem is the positive affirmation and acceptance of self-worth (48). Good self-esteem helps individual maintaining psychological balance and avoiding the psychological response caused by the pressure. Our results are consistent to the previous studies. A large number of studies showing that low self-esteem increases the risk of mental health problem, such as depression, anxiety (14, 49). In addition, low self-esteem was reported to be a risk factor for OCD, and enhancing self-esteem could significantly reduces OCD symptoms (50). Meanwhile, self-esteem played a mediating role in the relationship between left-behind experience and obsessive-compulsive symptoms (51). Too weak self-esteem is called inferiority, which would affect the self-evaluation, reduce social interaction, and increase interpersonal sensitivity (43). In the present study, we also found that low self-esteem increased the symptoms of interpersonal sensitivity, which are closely related to interpersonal relationships and social support. There are some evidence shows that low self-esteem is associated with interpersonal sensitivity. For example, Zavala et al. (52) reported that collective narcissism, a high but contradictory form of group self-esteem was associated with threats to group image and sensitivity to retaliatory aggression. And, Preti et al. (53) also found self-esteem was related to interpersonal relationships. Strong evidence shows that interpersonal relationships and social support are closely related to mental health (54). Good interpersonal relationships and social support reduce the incidence of mental health problems, otherwise increase the risk. A recent review reported by Gilligan et al. (55) concluded that the interventions on improving interpersonal communication skills among medical students had positive effects on most outcomes in most studies.

Moreover, we also found cognitive fusion and empirical avoidance are positively associated with obsessive-compulsiveness, interpersonal sensitivity, or depression. That means psychological flexibility was a protective factor for these three psychological symptoms. Studies have shown that the levels of cognitive fusion and empirical avoidance in patients with depression are higher than in health control group, and both psychological inflexibility are positively associated with depression level (56). Acceptance and commitment therapy (ACT) is a psychological intervention that aims at increasing psychological flexibility (22). Extensive evidence shows that ACT can improve depression, stress, and physical pain (23–25). Some studies shows that ACT can reduce OCD symptoms (57). Such as a randomized controlled trial study reported that SSRI combined with ACT treatment showed a greater decrease in the severity of OCD, compared with OCD patients with SSRI alone (58). However, researches on the relationship of ACT or psychological flexibility and interpersonal sensitivity are very lack. Oró et al. found mindfulness-based programme could significantly improve the symptoms of compulsion, interpersonal sensitivity of medical students (59). Our study might be the first study to report that interpersonal sensitivity was positively associated with psychological inflexibility. We speculated that enhancing the psychological flexibility by ACT or mindfulness would be helpful to reduce the symptoms of interpersonal sensitivity and increase social interaction.

The bootstrap method further showed that psychological flexibility played a partial mediating role between the self-esteem and the three most common psychological symptoms among medical students. The proportion of indirect effects fluctuated from 45.14 to 61.36%, which means that approximately 50% effects of self-esteem on the three most common psychological symptoms exert indirect effects through psychological flexibility. Empirical avoidance accounted for a higher proportion of indirect effects compared with cognitive fusion. Individuals with good self-esteem are more likely to recognize their abilities, objectively evaluate their value, and actively seek resources and methods to resist negative factors in the face of failure (60). However, the individuals with low self-esteem are prone to fall into rumination, become self-derogatory, and avoid solving problems when faced with pressure or conflicts (61). These manifestations are associated with psychological flexibility and psychological inflexibility, respectively (62). Similarly, according to the ACT treatment model, improving the level of the openness and acceptance might also improve the level of self-efficacy, which is positively associated with self-esteem (63). These theories support the finding that psychological flexibility might be a mediator between self-esteem and mental health. Therefore, we speculated that ACT intervention might be one of the effective methods to reduce the impact of low self-esteem on the mental health among medical students.

There were some limitations in the present study. Firstly, this study just used experiential avoidance and cognitive fusion to assess psychological flexibility. Future studies might use multidimensional measurement tools to comprehensively reflect the psychological flexibility of participants. Secondly, the participants enrolled in the study were from the same medical university. Thus, our finding might be more likely to reflect the prevalence of psychiatric symptoms among medical students in a region. Last, the study was a cross-sectional survey, so the causal relationship between variables could not be proven. Therefore, longitudinal follow-up study could be performed to verify the mediating effect of psychological flexibility on self-esteem and the symptoms of obsessive-compulsive disorder, interpersonal sensitivity and depression in the following studies.

## Conclusion

We found medical students displayed a considerable prevalence of psychopathological symptoms. Among the nine dimensions of psychological symptoms assessed by SCL-90-R, obsessive-compulsive was most prevalent, followed by interpersonal sensitivity and depression in the medical students. In addition, we identified a number of factors associated with the top three symptoms, factors including age, sex, grades, roommate relationship, family economic status, self-esteem, experiential avoidance and cognitive fusion. Furthermore, we found that psychological flexibility is a mediator in the relationship between self-esteem and mental health. We argue it is time to comprehensive identification of the mental health problems among medical students, followed by assessment-based psychological interventions to promote mental health.

## Data Availability Statement

The original contributions presented in the study are included in the article/supplementary material, further inquiries can be directed to the corresponding author.

## Ethics Statement

The study was approved by the Ethics Committee of the First Affiliated Hospital of Chongqing Medical University (2019-270). The participants provided their written informed consent to participate in this study.

## Author Contributions

JG and XH analyzing the data and writing the paper. AZ helping modifying the paper. WC helping designing the questionnaire. ZL, CT, HC, and HM helping collecting the data. XL: conceived and designed the experiments; fund provider; writing—review. All authors contributed to the article and approved the submitted version.

## Funding

This work was supported by Teaching Research Project of the First Affiliated Hospital of Chongqing Medical University (CMER201803), the Humanities and Social Sciences General Project of Chongqing Municipal Education Commission (17SKG023), the Chongqing Science and Health Joint Medical Scientific Research Project (2020MSXM079) and Teaching Reform Project of Hunan University of Medicine (2021JG23).

## Conflict of Interest

The authors declare that the research was conducted in the absence of any commercial or financial relationships that could be construed as a potential conflict of interest.

## Publisher's Note

All claims expressed in this article are solely those of the authors and do not necessarily represent those of their affiliated organizations, or those of the publisher, the editors and the reviewers. Any product that may be evaluated in this article, or claim that may be made by its manufacturer, is not guaranteed or endorsed by the publisher.

## Abbreviations

SCL-90-R: Symptom Checklist-90-revised
SES: Self-esteem Scale
AAQ-II: Acceptance and Action Questionnaire 2nd Edition
CFQ-F: Cognitive Fusion Questionnaire
ACT: Acceptance and Commitment Therapy
NSSI: non-suicidal self-injury (NSSI)
Y-BOCS: Yale-Brown obsessive compulsive scale(Y-BOCS)
OCD: obsessive-compulsive disorder.
